# Understanding the shortcomings of heart rate variability as a tool for autonomic analysis

**DOI:** 10.3389/fphys.2026.1760160

**Published:** 2026-01-26

**Authors:** Arijita Banerjee

**Affiliations:** Physiology, IIT Kharagpur, Kharagpur, India

**Keywords:** autonomic nervous system, frequency domain analysis, heart rate variability (HRV), limitations and barriers, machine learning (ML), time domain analysis

## Introduction

Heart rate variability (HRV) represents the physiological variation in the time interval between consecutive heartbeats, reflecting the complex interplay between the autonomic nervous system and cardiovascular regulation. Over the past several decades, HRV has emerged as a non-invasive biomarker with applications spanning clinical cardiology, mental health assessment, athletic performance optimization, and stress management. The measurement quantifies the beat-to-beat alterations in heart rate, primarily mediated by the dynamic balance between sympathetic and parasympathetic nervous system activity. While a perfectly regular heartbeat might seem ideal, healthy cardiovascular systems actually exhibit considerable variability, with reduced HRV often indicating physiological stress, disease states, or compromised autonomic function. This narrative review examines the multifaceted advantages and inherent limitations of HRV assessment while exploring how contemporary machine learning methodologies are revolutionizing the field by addressing traditional pitfalls and unlocking new clinical and research applications (Li et al., 2019; Shaffer and Ginsberg, 2017).

## Contributions of heart rate variability in autonomic neuroscience

Heart rate variability (HRV) is a fully non-invasive, accessible, and cost-effective measure of cardiovascular and autonomic function, obtainable through ECG or wearable PPG devices for continuous daily and clinical monitoring. It captures sympathetic–parasympathetic dynamics, providing a unique window into autonomic nervous system health. In cardiovascular medicine specifically, diminished HRV serves as an independent predictor of mortality following myocardial infarction and correlates with heart failure deterioration. Low HRV values indicate elevated risk for coronary artery disease and stroke. The reduction in HRV parameters reflects decreased parasympathetic activity, which directly correlates with heightened mortality risk and increased arrhythmogenic susceptibility (Molina et al., 2016; Banerjee A et al., 2023).

Beyond cardiovascular applications, HRV has illuminated autonomic dysfunction in neurodegenerative conditions. In glaucoma, emerging evidence suggests that reduced HRV may reflect dysregulation of ocular blood flow autoregulation, potentially linking systemic autonomic dysfunction to optic nerve damage progression. This represents a paradigm shift in understanding glaucoma beyond purely intraocular pressure mechanisms. In peripheral and autonomic neuropathies, HRV assessment provides objective quantification of autonomic nerve damage. Diabetic neuropathy research extensively utilizes HRV testing to detect subclinical autonomic involvement before symptom onset. Similarly, in conditions like Parkinson’s disease and multiple system atrophy, altered HRV patterns help differentiate between neurodegenerative syndromes and monitor disease progression. In mental health, it reflects stress and emotional regulation, supporting diagnosis and biofeedback-based interventions (Reyes del Paso et al., 2013; Banerjee and Khurana, 2017). The technique’s accessibility and reproducibility make HRV an invaluable research and clinical tool, bridging autonomic neuroscience with practical patient management across multiple disease states, ultimately enhancing our understanding of systemic autonomic integration in health and disease.

## Disadvantages and limitations of heart rate variability

### Measurement standardization challenges

Despite widespread adoption, HRV assessment suffers from significant standardization problems that limit clinical utility and research comparability. Numerous measurement protocols exist, varying in recording duration, body position, time of day, breathing control, and pre-measurement activities. Short-term recordings (5 min) provide different information than ultra-short recordings (1 min) or 24-h ambulatory recordings. Time-domain metrics like SDNN (standard deviation of NN intervals) calculated from 5-min recordings differ substantially from 24-h SDNN values, yet both are commonly reported. Frequency-domain analysis requires specific recording conditions and signal processing parameters that vary across studies. This lack of standardization means that reference values from one study may not apply to measurements obtained under different conditions, complicating clinical interpretation and limiting meta-analyses. Furthermore, different HRV software packages may produce different results from identical data due to varying algorithms for artifact correction and analysis (Camm et al., 1996).

### Differences between short- and long-term HRV arise because one measures regulated conditions, whereas the other mirrors lived physiology

HRV assessment is divided into short-term and long-term analyses based on recording duration. Short-term HRV, typically derived from 5-min segments, relies mainly on spectral components to gauge autonomic function under controlled conditions. Long-term HRV, usually measured over 24 h using wearable devices, incorporates time-domain, frequency-domain, nonlinear, and signal-averaged metrics, and is primarily used for evaluating mortality risk. Beyond duration, the key distinction lies in the measurement context: short-term recordings occur in standardized settings, whereas long-term recordings reflect real-life activity. Recent findings show that 24-h SDNN is strongly influenced by day–night heart rate variation, which correlates with daytime physical activity levels—implying that low SDNN may reflect limited activity due to underlying illness. Although LF/HF rises during standing, long-term studies have paradoxically linked reduced LF/HF to higher mortality, suggesting that its prognostic meaning differs from acute autonomic responses. Although HRV is linked to numerous health outcomes, applying these insights in clinical practice remains difficult. Clear intervention strategies for abnormal HRV are lacking, and most guidelines do not specify how HRV should guide treatment. Because HRV fluctuates naturally, identifying meaningful changes versus normal variation requires repeated measurements and careful clinical interpretation (Hayano et al., 1990; Kiyono et al., 2008).

### Sensitivity to artifacts and signal quality

HRV measurement is extremely sensitive to artifacts, ectopic beats, and signal quality issues that can substantially distort results. Premature ventricular contractions, atrial fibrillation episodes, or even occasional missed beats dramatically alter HRV metrics. While algorithms exist for artifact detection and correction, they imperfectly distinguish true physiological variations from measurement errors. In populations with frequent arrhythmias, HRV assessment becomes problematic or impossible. Motion artifacts during ambulatory monitoring corrupt signals, particularly with PPG-based measurements from wearable devices. Even breathing pattern variations during measurement significantly influence HRV, especially high-frequency components, yet spontaneous breathing is rarely controlled in clinical settings. Poor electrode contact in ECG recordings or inadequate perfusion in PPG measurements produces unreliable data. These technical challenges require careful quality control and expert interpretation, limiting HRV’s utility for automated, unsupervised monitoring in many contexts (Stein et al., 2008).

### Complex interpretation and physiological specificity

Interpreting HRV results presents significant challenges because multiple physiological and non-physiological factors influence measurements. While low HRV generally indicates poor health, the specific mechanisms remain unclear in individual cases. Reduced HRV might reflect increased sympathetic activity, decreased parasympathetic activity, or both, and distinguishing between these scenarios requires additional testing. Age profoundly affects HRV, with progressive declines throughout life, necessitating age-specific reference ranges that remain poorly defined for many populations. Sex differences exist, with women generally showing different HRV patterns than men. Medications, particularly beta-blockers and anticholinergics, dramatically alter HRV independent of disease status. Physical fitness, body composition, and genetic factors contribute substantial inter-individual variability, making it difficult to establish universal normal ranges. The non-specific nature of reduced HRV means it signals that something may be wrong without indicating what specifically is problematic (Stein et al., 2005).

### HRV captures only the neural regulation of the heart

Heart rate variability (HRV) reflects autonomic signals that act specifically on the sinoatrial node, since its fluctuations arise from brain-generated inputs transmitted through cardiac sympathetic and vagal fibers. Therefore, HRV represents only the autonomic control of cardiac pacemaking, not the autonomic status of the entire body. Autonomic responses in other organs may differ substantially; for instance, digestion increases vagal activity in the gastrointestinal system while simultaneously reducing cardiac vagal outflow to raise heart rate. While sympathetic regulation across organs may show some parallel patterns, parasympathetic activity is highly organ-specific. As a result, HRV cannot be used to infer parasympathetic functions outside the heart (Kumar and Banerjee, 2023).

### Respiratory sinus arrythmia and mayer wave sinus arrythmia (MWSA)

The distinction between sympathetic and vagal frequency bands in HRV is often overstated, as the presence of high-frequency components merely indicates vagal mediation of fluctuations—not the overall level of cardiac vagal tone. HF power can decrease simply because breathing rate falls outside the HF range, making it an unreliable standalone marker of vagal withdrawal. Because multiple physiological processes contribute to HRV, linking frequency bands directly to autonomic divisions is overly simplistic; instead, HRV should be interpreted as the sum of distinct mechanisms, primarily respiratory sinus arrhythmia and the ∼0.1 Hz Mayer wave fluctuations driven by baroreflex activity (Brown et al., 1993; Sakakibara et al., 2008).

### Limitations of consumer devices

The explosion of consumer wearable devices offering HRV monitoring presents both opportunities and significant concerns. While democratizing access to HRV data, most consumer devices use PPG technology rather than ECG, which is less accurate for HRV assessment. PPG sensors are more susceptible to motion artifacts and produce less precise inter-beat interval measurements. Many devices employ proprietary algorithms and provide only simplified HRV metrics or arbitrary scores rather than standard clinical parameters, preventing comparison with research literature or clinical assessments. Validation studies frequently show poor agreement between consumer devices and gold-standard ECG measurements, particularly during movement or in individuals with dark skin where PPG signal quality degrades. Users may make inappropriate health decisions based on unreliable data, and the clinical significance of metrics reported by consumer devices remains unclear. The lack of regulatory oversight for many wellness devices means accuracy claims may not undergo rigorous independent verification (Banerjee, 2025).

### Machine learning approaches to overcome the limitations of traditional HRV

#### Automated artifact detection and signal quality assessment

Machine learning models, particularly deep learning architectures, identify artifacts and assess signal quality far more accurately than traditional threshold-based methods. These systems can detect, classify, and even correct corrupted intervals in real time, greatly improving the reliability of HRV measurements. Deep learning approaches, particularly convolutional neural networks (CNNs) and recurrent neural networks (RNNs), can learn complex patterns distinguishing artifacts from genuine physiological signals. These models train on large datasets of labelled ECG recordings where artifacts are identified by experts, then generalize to automatically detect similar patterns in new data as shown in Table 1. Long short-term memory (LSTM) networks excel at sequence modelling tasks, making them ideal for identifying context-dependent artifacts that appear normal in isolation but anomalous given surrounding beats (Persson et al., 2020; Liang et al., 2020).

**TABLE 1:** Recent studies incorporating machine learning and artificial intelligence techniques to analyze HRV data.

Author and year	Machine learning method used	HRV parameters	Findings
Rosenberg et al. (2017)	2D scatter plot analysis	LF, HF, LF/HF ratio, LFn, HFn, LFp, HFp, LFiA, HFiA	2D scatter plots achieved 90% accuracy for stress detection, superior to 1D methods (70% accuracy)
Wang et al. (2018)	SVM, KNN	R-R interval, LF/HF, SDNN	100% accuracy in distinguishing CHF from normal sinus rhythm
He et al. (2019)	CNN (Convolutional Neural Network)	ApEn, LF, PSD features (0.04–20 Hz)	17.3% error rate for stress detection using ultra-short 10s windows
Castaldo et al. (2019)	TPOT (automated ML), SVM, MLP, IBK, C4.5, LDA	MeanNN, stdHR, HF (ultra-short term features)	Successfully classified stress using ultra-short term HRV features in real-time
Lima et al. (2019)	Random Forest, SVM	HR, RR-interval, SD1/SD2, SCL, SCR	80% accuracy using RF for stress classification; 77% with EDA features
Posada et al. (2019)	KNN, Linear SVM, LDA	SCL, TVsym, LFn	66%–69% accuracy for cognitive task classification; accuracy declined after 20h wakefulness
Cho et al. (2019)	CNN	Raw ECG signals	90.19% accuracy for stress detection from raw ECG
Coutts et al. (2020)	LSTM (Long Short-Term Memory)	Time-domain HRV features	83% accuracy for mental health prediction
Taye et al. (2019)	ANN (Artificial Neural Network)	QRS complex features	98.6% accuracy; 26.6% improvement using novel feature engineering
Arsalan et al. (2019)	MLP (Multi-Layer Perceptron)	EEG and HRV features	92.85% accuracy for mental stress classification
Ma et al. (2020)	CNN, MLP	HRV features for atrial fibrillation	96.58% (CNN), 98.2% (MLP) accuracy
Persson et al. (2020)	KNN, SVM, AdaBoost, Random Forest	Time and frequency domain features	77.5% (KNN), 83.4% (SVM), 82.4% (AdaBoost), 85.4% (RF) for drowsiness detection while driving
Liang et al. (2020)	CNN with bidirectional long short-term memory (BiLSTM)	HRV features from processed ECG	Classification accuracy of cardiac beats (96%)

#### Personalized HRV baselines and trajectories

Unsupervised learning techniques like clustering and principal component analysis identify HRV phenotypes within populations, recognizing that different individuals may have distinct healthy HRV patterns. Some people naturally exhibit lower HRV without adverse health consequences, while others with moderate values face elevated risk. Machine learning classification models trained on individual characteristics (age, sex, fitness, genetic markers) predict expected HRV ranges, improving interpretation accuracy (Cho et al., 2019; Coutts et al., 2020; Posada et al., 2019).

#### Integration of multimodal data

Machine learning integrates HRV with complementary physiological and clinical data, enhancing diagnostic and prognostic accuracy beyond HRV alone. Multimodal fusion reveals contextual relationships that clarify the interpretation of HRV changes across diverse conditions. Convolutional neural networks analyzing raw ECG waveforms often extract features beyond conventional HRV metrics that improve diagnostic and prognostic performance. In mental health applications, combining HRV with actigraphy, speech patterns, and smartphone usage data enhances depression and anxiety prediction (Castaldo et al., 2019; Lima et al., 2019).

#### Advanced feature extraction and non-linear analysis

Deep learning and nonlinear methods extract complex HRV features that reflect physiological dynamics beyond traditional time and frequency metrics. Non-linear dynamics measures like approximate entropy, sample entropy, and detrended fluctuation analysis capture HRV complexity that time and frequency domain metrics miss, but their calculation and interpretation present challenges. Machine learning clarifies the clinical significance of these complex measures by relating them to outcomes in large datasets. These advanced representations improve prediction accuracy, especially in non-stationary conditions like exercise or stress testing (Rosenberg et al., 2017; Wang et al., 2018; He et al., 2019).

#### Standardization through transfer learning

Transfer learning enhances consistency across devices and protocols by creating shared model representations that generalize well to diverse HRV data sources. Domain adaptation and federated learning further reduce variability while maintaining data privacy. Models trained on gold-standard ECG recordings can be adapted to work with PPG measurements from wearables through domain adaptation techniques. Adversarial training creates device-agnostic HRV features that capture physiological signals while being insensitive to device-specific artifacts and measurement characteristics (Banerjee, 2025).

## Challenges of using machine learning in heart rate variability analysis

Machine learning applications in HRV analysis face multiple critical challenges. Data quality issues including artifacts, noise, and lack of standardization across devices compromise model reliability. Small sample sizes, class imbalance, and limited demographic diversity hinder generalization. The high-dimensional feature space and temporal dependencies require sophisticated approaches, while deep learning’s “black box” nature limits clinical interpretability and trust. HRV’s sensitivity to numerous physiological confounders like age, medications, stress, circadian rhythms complicate accurate disease attribution. Additionally, rigorous clinical validation for regulatory approval and privacy concerns surrounding continuous health monitoring present significant barriers to successful translation from research to real-world clinical implementation (Banerjee J. S et al., 2023).

## Future directions and conclusion

The integration of machine learning with HRV assessment represents a paradigm shift in cardiovascular and autonomic monitoring. While HRV offers tremendous advantages as a non-invasive window into autonomic function and cardiovascular health, traditional limitations around standardization, interpretation, and clinical applicability have restricted its adoption. Machine learning directly addresses these challenges through improved signal processing, personalized baselines, multimodal integration, automated feature discovery, enhanced prediction, and real-time decision support.
